# Antimicrobial Resistance Among Selected Zoonotic Bacteria: A Case Study of Tortoises and Birds at the Uganda Wildlife Conservation Education Centre

**DOI:** 10.1002/vms3.71157

**Published:** 2026-08-10

**Authors:** Delilah Diana Namara, Paul Ssajjakambwe, Sarah Robinah Nakabuye, Vanessa Alyj Katabazi, Celsus Sente

**Affiliations:** ^1^ College of Veterinary Medicine Animal Resources and Biosecurity Makerere University Kampala Uganda; ^2^ Faculty of Health Sciences University of Cape Town Cape Town South Africa; ^3^ National Agricultural Research Organisation Kampala Uganda; ^4^ Bukalasa Agricultural College Wobulenzi Uganda; ^5^ Animal Allies Africa Masaka Uganda

**Keywords:** antimicrobial resistance, wildlife, zoonoses

## Abstract

**Background:**

Zoonotic diseases are a health challenge at the human–wildlife interface with increasing threat to public health. This study aimed at generating baseline data on antimicrobial resistance (AMR) in tortoises and birds at the Uganda Wildlife Conservation Education Centre (UWEC).

**Objectives:**

The objectives of the study were determining the common antimicrobial‐resistant bacteria among birds and tortoises, to establish the antibiograms of isolated bacteria and lastly to genotypically screen the selected genes that code for resistance using a conventional polymerization chain reaction.

**Methods:**

Standard bacteriological methods of culturing and isolation of bacteria, Kirby–Bauer sensitivity method and molecular techniques were used at the Central Diagnostic Laboratory at the College of Veterinary Medicine, Animal Resources and Biosecurity at Makerere University.

**Results:**

*Escherichia coli* was isolated from all the 23 bird samples, 23/23 (100%). In the tortoises, 2/15 (13%) and 13/15 (86%) isolates were *E. coli* and *Salmonella enteritidis*, respectively. *E. coli* showed equally higher resistance to ampicillin and SXT (68%), whereas *S. enteritidis* exhibited resistance to only ciprofloxacin (30.8%). Sul‐2 resistance genes were detected in 52% of *E. coli* and 15.4% of *S. enteritidis* isolates.

**Conclusion:**

The UWEC management and workers are encouraged to take care and use personal protection equipment when handling the birds and tortoises to minimize the possibility of transfer of MDR isolates from animals to humans.

## Graphical abstract

This study investigated antimicrobial resistance in birds and tortoises at the Uganda Wildlife Conservation Education Centre. *Escherichia coli* predominated in birds, whereas *Salmonella enteritidis* predominated in tortoises with resistance to sulphonamides detected. The findings highlight potential zoonotic risks and emphasize using personal protective equipment during wildlife handling.

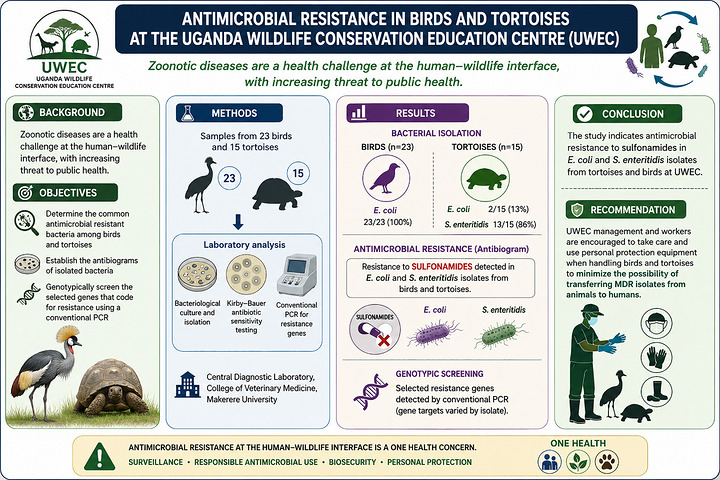

## 1 Introduction

Antimicrobial resistance (AMR) is an emerging and rapidly growing public and animal health threat, arising when microbes develop mechanisms to evade the effects of antimicrobial agents (Tenover 2006; Opintan et al. 2015). Globally, AMR contributes to approximately 700,000 deaths annually, and projections indicate that without urgent intervention, previously manageable infections and minor injuries may once again become fatal (Clifford et al. 2018; Ampaire et al. 2016). The economic consequences are substantial, with illnesses caused by resistant bacteria costing an estimated $4–$5 billion per year, currently reducing global GDP by 0.4%–1.6%, and potentially reaching a 3.5% reduction by 2050, amounting to $100 trillion (Kilonzo‐Nthenge et al. 2008; Barbhuiya et al. 2019). Surveillance and monitoring are globally recognized as critical components of the response to AMR, enabling early detection, identification of at‐risk populations, informed policy‐making and guidance for clinical and veterinary treatment (Opintan et al. 2015). However, in many developing countries, including those in Africa, effective surveillance is constrained by weak legal and regulatory frameworks, limited laboratory capacity, poor data sharing and insufficient inter‐sectoral coordination (Clifford et al. 2018). In East Africa, AMR surveillance remains insufficient, and data on the prevalence, burden and transmission pathways of resistant pathogens are scarce (Opintan et al. 2015). In Uganda specifically, there is currently no organized surveillance system for AMR in animals, highlighting an urgent need to generate baseline information to guide national policy and One Health interventions.

Wildlife plays a crucial role in the emergence and maintenance of zoonotic pathogens, many of which are bacterial and capable of developing AMR. More than 60% of human pathogens are of zoonotic origin, and most emerging infectious diseases are linked to wildlife reservoirs (Rahman et al. 2020). In Africa, human–wildlife–livestock interfaces are widespread due to poaching, open grazing, urban expansion and encroachment into protected areas, creating frequent opportunities for pathogen spillover (Hassell et al. 2017). Resistant *Salmonella* and *Escherichia coli* have been reported in wild birds and reptiles, underscoring the role of wildlife as reservoirs and potential dispersers of resistant bacteria (Pees et al. 2023). In Uganda, resistant *E. coli* and *Salmonella* have been isolated from livestock, humans, and animal‐source foods, highlighting cross‐species circulation of resistant pathogens (Manishimwe et al. 2021). A study in Wakiso district found poultry farms to harbour multidrug‐resistant *Salmonella* and pathogenic *E. coli*, whereas wild birds that forage in landfills and sewage‐contaminated water were found to introduce resistant bacteria into food and water systems (Ssemakadde et al. 2025; Bonnedahl and Järhult 2014). Although Uganda's national zoonotic disease prioritization exercise identified anthrax, brucellosis and viral haemorrhagic fevers as top threats, bacterial zoonoses with AMR potential remain underexplored (Sekamatte et al. 2018).

Given these challenges, there is a critical need for integrated One Health research to understand the occurrence and resistance patterns of zoonotic bacteria at human–wildlife interfaces in Uganda. Therefore, this study aimed to investigate the prevalence and AMR of zoonotic bacteria in tortoises, birds and humans at the Uganda Wildlife Conservation Education Centre (UWEC), providing baseline data to inform national surveillance strategies, guide policy decisions and contribute to mitigating the growing AMR threat in Uganda.

## 2 Materials and Methods

### 2.1 Study Area

**FIGURE 1:**
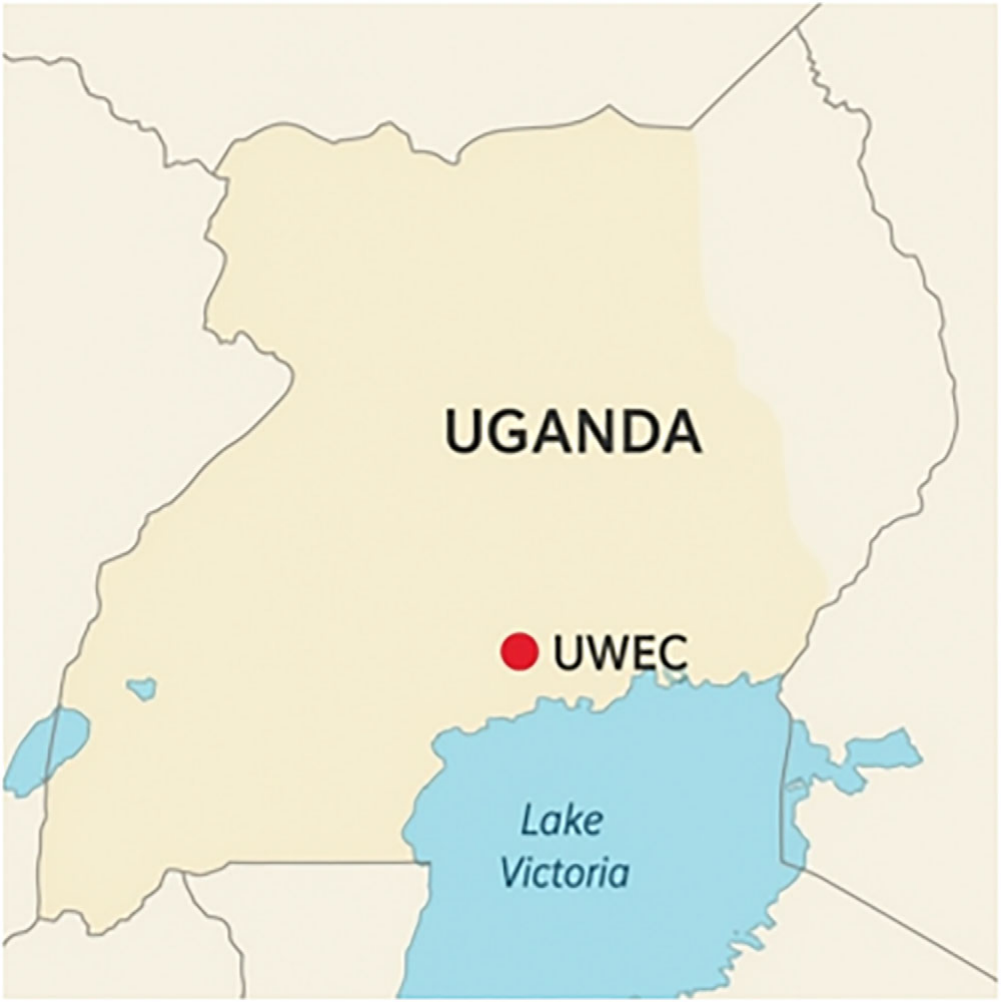
Map showing location of Uganda Wildlife Conservation Education Centre (UWEC).

The study was done at the UWEC which is a zoo in central Uganda in Wakiso district, Entebbe municipality that houses different animal species. UWEC (Figure 1) is located at 0.05294° N latitude and 32.47605° E longitude. It has an elevation of 1148 m at 0.0533° N and 32.4764° E in Manyango village. It was originally founded in the 1950s to accommodate confiscated and injured wildlife and to look after orphaned animals confiscated from smugglers. This site was chosen because humans are in close and constant contact with the animals, thus having higher chance of dissemination of micro‐organisms, including those carrying resistant genes.

### 2.2 Sample Collection

This was a cross‐sectional study that investigated AMR among selected zoonotic bacteria in tortoises, birds and humans at UWEC. Purposive sampling was conducted on the basis of availability and adherence to the conditions provided for sample collection. The species sampled are summarized in Table 1.

**TABLE 1:** Birds and tortoise species sampled.

Scientific name	Common name
*Balaeniceps rex*	Shoebill stork
*Pavo cristatus*	Indian peafowl
*Gallus gallus domesticus*	Domestic chicken
*Meleagris gallopavo* f. *domestica*	Domestic turkey
*Numida meleagris*	Helmeted guinea fowl
*Balearica regulorum*	Grey‐crowned crane
*Psittacus erithacus*	African grey parrot
*Anser anser*	Domestic Emden goose
*Struthio camelus*	African ostrich
*Kinixys belliana*	Bell‐hinged tortoise
*Stigmochelys pardalis*	Leopard tortoise

The study involved the determination of AMR in priority zoonotic bacteria, namely, *E. coli*, *Salmonella* spp. and *Staphylococcus aureus*. Samples collected from tortoises and birds included faecal matter, cloacal swabs and oropharyngeal swabs, whereas human samples were collected from the palmar and dorsal surfaces of the hands of zoo caretakers using sterile swabs. Antimicrobial susceptibility testing of the isolates was performed, followed by genotypic screening for selected resistance genes from phenotypically resistant isolates using conventional polymerase chain reaction (PCR).

Tortoises were physically restrained and placed on dorsal recumbency, and a mouth gag was used to access the oral cavity for oropharyngeal sampling according to Perpiñán (2017). Both oropharyngeal and cloacal samples were collected from tortoises, and faecal samples (2–5 g) were obtained from fresh environmental faecal matter. Birds were also physically restrained, and oropharyngeal and cloacal samples were collected along with faecal samples (2–5 g) from fresh environmental faecal matter.

Following collection, samples were placed in aseptically clean cryogenic vials containing buffered peptone water using gloved hands and sterilized equipment, including mouth gags and forceps, which were disinfected using 70% ethanol after each collection. The cryogenic vials were labelled with the sample ID, species and date of collection, then placed in a cool box containing ice packs and transported under cold chain conditions to the Central Diagnostic Laboratory (CDL) for bacteriology, antimicrobial susceptibility testing and PCR analysis.

All samples were collected aseptically using sterile swabs while wearing protective gloves and face masks to minimize contamination. In the laboratory, media quality was ensured by following the manufacturer's instructions, and all tests were performed in a Class II biosafety cabinet to prevent contamination. Positive controls were used for biochemical tests, and McFarland turbidity standards were applied to ensure appropriate turbidity of bacterial suspensions during sensitivity testing.

### 2.3 Culture and Identification of Bacteria

All samples were initially enriched by incubation in buffered peptone water for 24 h at 37°C. Buffered peptone water was prepared according to the manufacturer's instructions (16.1 g suspended in 1000 mL of distilled water). Following enrichment, selective culture was performed to enhance recovery of target organisms.

For isolation of *S. aureus*, enriched swabs were inoculated into 7% NaCl nutrient broth and incubated at 37°C for 24 h. For *Salmonella* spp. isolation, 0.1 mL of the enriched culture was transferred into 2 mL of Rappaport‐Vassiliadis broth and incubated at 42°C for 24 h. Selective and differential media used included MacConkey agar, *Salmonella*–*Shigella* (SS) agar and mannitol salt agar. All inoculated media were incubated at 37°C for 24 h. After incubation, distinct colonies were selected and subcultured to obtain pure isolates.

Bacterial identification was based on colony morphology, Gram staining and standard biochemical tests. The media and enrichment procedures were selected to enhance the isolation of *E. coli*, *Salmonella* spp. and *S. aureus* in line with established protocols (Munshi et al. 2012; Maddocks et al. 2002; Jahan et al. 2014).

### 2.4 Antimicrobial Sensitivity Test (Antibiograms)

Antimicrobial susceptibility testing (antibiograms) was performed using the Kirby–Bauer disk diffusion method on Mueller–Hinton agar (MHA) plates (Biemer 1973). A standardized bacterial suspension equivalent to 0.5 McFarland standard (approximately 1.5 × 10^8^ CFU/mL) was prepared in tryptone soya broth and used to inoculate the agar surface.

Using a sterile cotton swab, the bacterial suspension was evenly streaked over the entire surface of the MHA plate in three directions, rotating the plate approximately 90° between streaking to ensure uniform confluent growth. The swab was also passed along the edges of the plate to ensure complete coverage. Antibiotic discs (Table 2) were aseptically placed on the inoculated agar surface using sterile forceps and spaced evenly apart to prevent overlapping zones of inhibition (Sawatzky et al. 2015). The plates were then inverted and incubated aerobically at 37°C for 16–18 h (CLSI 2017). After incubation, the diameters of the zones of inhibition were measured in millimetres using a ruler. The results were interpreted as susceptible (S), intermediate (I) or resistant (R) on the basis of standardized interpretive criteria provided by the disc manufacturer and the CLSI guidelines.

**TABLE 2:** Antimicrobial discs used.

Antimicrobial class	Antimicrobial discs	Concentration in µg/mL
Folate pathway inhibitors	Sulphamethoxazole Trimethoprim (SXT)	25
Quinolones	Ciprofloxacin (CIP)	30
Aminoglycosides	Gentamicin (CN)	10
β Lactams	Ampicillin (Am‐10)	10
Tetracyclines	Tetracycline (Te)	30
Carbapenems	Meropenem (MEM)	10

### 2.5 Screening for Resistance Genes

In this study a conventional PCR was run to screen for genes that code for tetracycline (*Tet M*) and sulphonamide (*Sul‐1*, *Sul‐2* and *Sul‐3*) resistances. The PCR reaction conditions used were; 2 µL of the crude DNA as template in 20 µL reaction volume, 10 µL of a 2X DreamTaq Green PCR Master Mix (which includes DreamTaq DNA Polymerase, 2X DreamTaq Green buffer, dNTPs and 4 mM MgCl_2_—Thermo Scientific), 2µL forward primer (5′‐AACTTAGGCATTCTGGCTCAC‐3′) and 2 µL reverse primer (5′‐TCCCACTGTTCCATATCGTCA‐3′), all primers were a 10 pmol/µL concentration. This reaction was carried out in a BIOBASE MINI PCR model DTC‐4C thermal cycler at 40 cycles, each consisting of 30 s at 94°C, 30 s at 55°C and 1 min at 72°C besides the initial denaturation step of 4 min at 94°C and the final extension at 72°C for 7 min. The band for this PCR was 515‐bp fragment detected after agarose gel electrophoresis.

### 2.6 Data Analysis

The collected data were recorded in prepared data sheets. Data were entered in MS Excel and exported to Rstudio (version R 4.5.1) for statistical analysis.

## 3 Results

### 3.1 Prevalence of AMR Priority Bacteria in Collected Samples

A total of 77 samples were collected from birds, tortoises and humans as summarized in Table 3. From these samples, 38/77 (49.35%) turned out positive for the pathogens of interest, and the rest were negative. Of the 38/77 (49.35%) samples, the overall samples that tested positive for *E. coli* were 25/77 (32.47%), and those that tested positive for *Salmonella enteritidis* were 13/77 (16.88%). Of these isolates, 23/38 (60.53%) were bird samples, and 15/38 (39.47%) were tortoise samples. All the 23 bird samples tested positive for *E. coli* 23/23 (100%), whereas for the tortoise samples, 2/15 (13.3%) tested positive for *E. coli* and 13/15 (86.67%) tested positive for *S. enteritidis*. The distribution of bacterial isolates differed significantly between birds and tortoises (*p* value <0.001). The prevalence of bacterial species by animal host is summarized in Table 4.

**TABLE 3:** Species and sample number.

Species	Samples	Sample type
Birds	49	Oropharyngeal swabs, cloacal swabs, environmental faecal sample
Tortoises	15	Oropharyngeal swabs, cloacal swabs, environmental faecal sample
Humans	13	Palm swab and backhand swab
Total	77	

**TABLE 4:** Bacteria in collected samples.

Bacterial species	Overall (*n* = 77)	Birds (*n* = 23)	Tortoises (*n* = 15)	*p* value^2^
*Escherichia coli*	25 (32.47%)	23 (92%)	2 (8%)	<0.05
*Salmonella enteritidis*	13 (16.88%)	—	13 (100%)	
Total	38 (49.35%)	23 (60.52%)	15 (39.47%)	

Of the 23 bird bacterial isolates, all of which were *E. coli*, 5/23 (21.74%) were cloacal swabs, 8/23 (34.78%) were faecal samples collected from the housing area, and 10/23 (43.48%) were oropharyngeal swabs. From the 15 tortoise bacterial isolates, 2/15 (13.33%) were *E. coli* isolates with 2/2 (100%) being oropharyngeal swabs, and from the 13/15 (86.67%) *S. enteritidis* isolates, 5/13 (38.46%) were cloacal swabs, 2/13 (15.38%) were faecal samples collected from the ground, and 6/13 (46.15%) were oropharyngeal swabs. The distribution of bacterial isolates by sample type is presented in Table 5.

**TABLE 5:** Bacteria in different sample types.

Animal species	Bacterial pathogen	Cloacal	Faecal	Oropharyngeal	Total
Birds	*Escherichia coli*	5/23 (21.74%)	8/23 (34.78%)	10/23 (43.48%)	23/23 (100%)
Tortoises	*Escherichia coli*	—	—	2/2 (100%)	2/2 (100%)
	*Salmonella enteritidis*	5/13 (38.46%)	2/13 (15.38%)	6/13 (46.15%)	13/13 (100%)

### 3.2 Antibiograms and Sensitivity Profiles of the Isolated Bacteria

**FIGURE 2:**
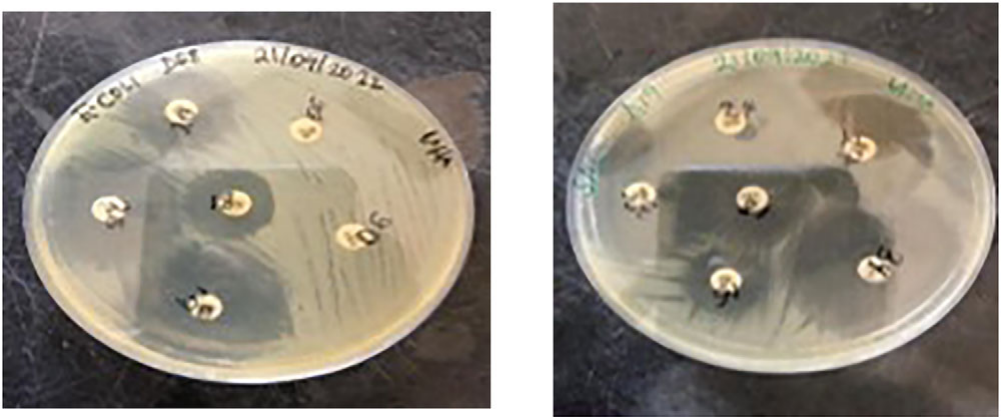
Sensitivity patterns for the different drugs.

All the 38 *E. coli* and *S. enteritidis* isolates were tested for antimicrobial susceptibility/resistance using 6 selected antibiotic discs. The overall sensitivity patterns for the tested antibiotics are shown in Figure 2.

**FIGURE 3:**
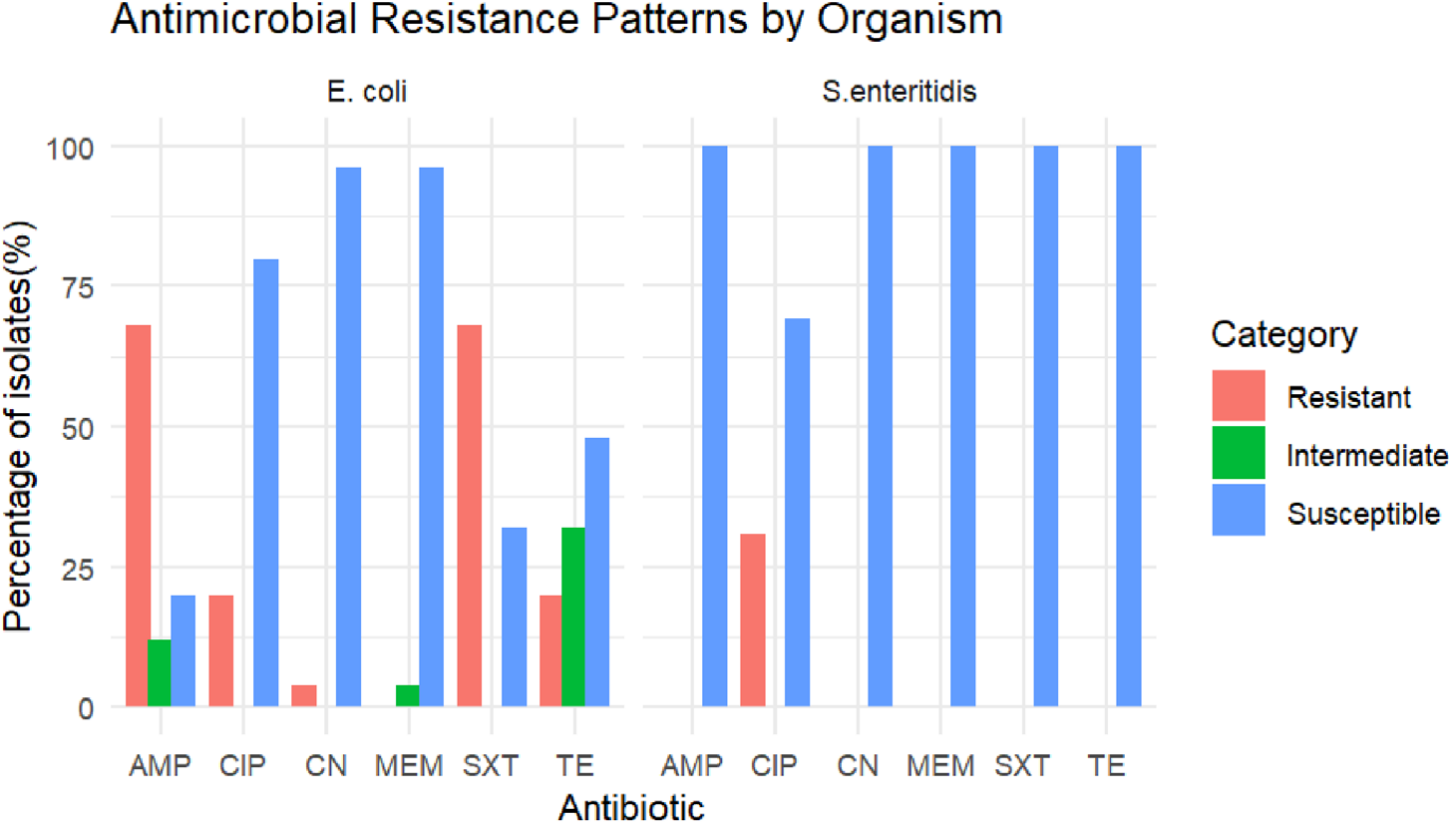
*Salmonella enteritidis* and *Escherichia coli* sensitivity patterns.

*S. enteritidis* exhibited resistance against only ciprofloxacin 4/13 (30.8%) with 13/13 (100%) susceptibility to all the other drugs. For *E. coli*, highest resistance was observed against ampicillin and sulphamethoxazole–trimethoprim 17/25 (68%) for both drugs. Subsequently, tetracycline and ciprofloxacin at 5/25 (20%). The best drugs of choice against it were meropenem 25/25 (100%) and gentamicin 24/25 (96%) followed by ciprofloxacin 20/25 (80%) and tetracycline 12/25 (48%) with ampicillin having the worst action against the bacteria 5/25 (20%) as shown in Figure 3.

### 3.3 Genetic Prevalence of Resistance Genes

**FIGURE 4:**
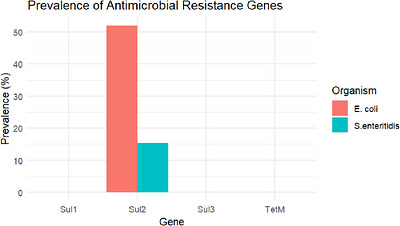
Genetic prevalence of resistant genes in *Escherichia coli* and *Salmonella enteritidis* isolates.

A conventional PCR was run to screen for genes that code for tetracycline (*Tet M*) and sulphonamide (*Sul‐1, Sul‐2* and *Sul‐3*) resistances. All isolates were negative for the *Tet M* gene that codes for tetracycline resistance. The same was noted for *Sul‐1* and *Sul‐3* genes; however, 2/13 (15.4%) *S. enteritidis* and 13/25 (52%) *E. coli* isolates were positive for *Sul‐2*. The distribution of detected resistance genes is illustrated in Figure 4.

## 4 Discussion

### 4.1 Prevalence of AMR Priority Bacteria in Collected Samples

This study area showed a statistically significant difference in distribution of isolates with high prevalence of *E. coli* in birds and high prevalence of *S. enteritidis* in tortoises. These findings highlight the widespread occurrence of enteric bacteria within captive wildlife populations and their potential role as reservoirs of antimicrobial‐resistant organisms.

The high prevalence of *E. coli* in faecal and cloacal samples from birds was expected because *E. coli* is a common commensal organism and normal flora inhabiting the gastrointestinal tract of birds. Its recovery from oropharyngeal swabs may be attributed to faecal‐oral contamination through feeding practices, particularly where feed is placed directly on enclosure surfaces that may be contaminated with faecal matter. Similar findings have been reported in Egypt (Biemer 1973), where *E. coli* was frequently isolated from captive birds. However, lower prevalence estimates have been reported elsewhere (Merwad et al. 2014), possibly due to differences in sample collection methods, environmental conditions, management systems and bacterial isolation procedures.

Among tortoises, the detection of *E. coli* in oropharyngeal swabs may be due to cross‐contamination between faeces and the water in which tortoises spend a lot of time and drink. Indirect transmission through handlers may also contribute to bacterial circulation within captive environments.

*S. enteritidis* was not detected among birds in this study. This finding is consistent with reports from Canada (Merwad et al. 2014) and the United States, where *Salmonella* was not detected in certain captive bird populations. In contrast, studies conducted in the Czech Republic (Vogt et al. 2018) and other regions have reported detectable *Salmonella* prevalence. Differences between studies may be attributed to variations in husbandry practices, feed contamination, environmental hygiene, sample handling procedures and the degree of human–animal interaction (Konicek et al. 2016; Hanson 2002).

The prevalence of *S. enteritidis* observed among tortoises was probably because *Salmonella* is part of the normal flora reptile gut microbiota and its distribution can also vary due to various environments with captive tortoises observed with having higher shedding of *Salmonella* than wild ones mostly attributed to more stressors as well as type of feed. Variations in samples and methods employed for *Salmonella* spp. detection are also known to influence detection rates, considering that they can vary widely. This is important as this *Salmonella* species is commonly reported as one of the serovars responsible for human salmonellosis.

This was comparable to findings reported in Japan (Nowakiewicz et al. 2016; Santoro et al. 2007) and Spain (Shecho et al. 2017), where high carriage rates were documented. However, substantially lower prevalence estimates have been reported among gopher tortoises in Georgia (Kabiswa et al. 2018) and imported tortoises in Europe (Ramos et al. 2019). Such variations are common and may reflect differences in ecological conditions, animal management systems, geographic location and environmental exposure.

### 4.2 Antibiogram and Sensitivity Profiles of Isolated Bacteria

Antimicrobial susceptibility testing revealed the highest levels of resistance against ampicillin and sulphamethoxazole–trimethoprim among *E. coli* isolates. This could be as a result of historical or ongoing selective pressure associated with their frequent use in routine animal health management at UWEC, and thus, repeated exposure may promote the persistence and dissemination of resistant bacterial strains. Similar resistance patterns have been reported in Sri Lanka (Hidalgo‐Vila et al. 2007), where resistance to ampicillin and sulphamethoxazole–trimethoprim was frequently observed.

In contrast, resistance to gentamicin and meropenem was low. This observation may be explained by the limited use of these drugs due to their higher cost and their importance in the treatment of serious bacterial infections. The low resistance to gentamicin observed in this study is consistent with findings from Kenya (Hanson 2002), Nigeria (Sarba et al. 2019) and other studies (Bamunusinghage et al. 2022), where little or no resistance was detected among *E. coli* isolates. In contrast, significantly higher gentamicin resistance has been reported in Poland (Apata 2009), where more extensive therapeutic use was suggested as a contributing factor. Collectively, these findings support the notion that local antimicrobial usage practices play a major role in shaping resistance patterns within animal populations.

Resistance to ciprofloxacin in *Salmonella* isolates was probably due to usage of the drug in treatment and prophylactic antibacterial cover during routine animal health assessments. This contradicts studies, including one done in a zoological institute in Spain, where isolates had no resistance to the drug (Nowaczek et al. 2021), highlighting the geographical variability of AMR patterns. Such differences may be influenced by variations in antimicrobial usage practices, regulatory policies, prescription habits and antimicrobial stewardship measures across regions.

### 4.3 Genetic Prevalence of Resistance Genes

Molecular characterization of selected resistance determinants revealed the absence of the *tetM*, *sul1* and *sul3* genes among all isolates screened. However, the *sul2* gene was detected in both *S. enteritidis* and *E. coli* isolates. The absence of *tetM* suggests that tetracycline resistance observed phenotypically may be mediated by alternative tetracycline resistance mechanisms or genes that were not included in this study. This finding highlights the genetic diversity of resistance mechanisms and underscores the importance of screening for multiple resistance determinants when investigating AMR.

The detection of *sul2* among both bacterial species suggests that this gene may be an important contributor to sulphonamide resistance within the study population. Sulphonamide resistance genes are commonly associated with mobile genetic elements which facilitate horizontal transfer between bacterial species and increase the potential for dissemination across different hosts and environments.

The occurrence of resistance genes in wildlife‐associated bacterial isolates is of particular concern from a One Health perspective. Wildlife populations are increasingly exposed to anthropogenic influences through environmental contamination, interactions with domestic animals and contact with humans. Consequently, resistant bacteria and resistance genes may circulate between humans, livestock, wildlife and the environment. The presence of *sul2* in isolates recovered from captive wildlife may therefore indicate environmental dissemination of resistance determinants across interconnected ecological systems (Ssajjakambwe et al. 2017).

This study provides important baseline evidence on AMR patterns and resistance genes among captive birds and tortoises in Uganda, a setting where such data remain scarce. A key strength lies in the integration of phenotypic antimicrobial susceptibility testing with molecular detection of resistance genes which allowed for a more comprehensive characterization of resistance patterns than either approach alone.

Despite these strengths, several limitations should be acknowledged. The study was conducted within a single wildlife facility which may limit the broader generalizability of the findings to other captive‐ or free‐ranging wildlife populations. Nevertheless, the observed overlap in resistance profiles and shared presence of resistance genes across species highlights the likely influence of interconnected ecological systems in shaping AMR dynamics. This underscores the importance of interpreting these findings within a One Health framework, where humans, animals and the environment are considered as part of a single epidemiological continuum.

Future research should therefore expand beyond facility‐based sampling to include environmental reservoirs, such as water, feed and enclosure surfaces, which may act as important sources or transmission pathways of resistant organisms. Additionally, larger multi‐site studies incorporating a broader range of wildlife species would improve generalizability and allow for more robust comparisons across ecological settings.

## 5 Conclusion

The study showed statistically significant difference in distribution of bacterial isolates with a high prevalence of *E. coli* in birds and *S. enteritidis* detected only in tortoises at UWEC. This indicates widespread circulation of enteric bacteria within captive wildlife populations.

AMR was observed in both *E. coli* and *S. enteritidis* isolates, with the highest resistance recorded for ampicillin (68%) and sulphamethoxazole–trimethoprim (44.74%). The detection of the *sul2* gene confirms the genetic basis of sulphonamide resistance in the study isolates.

These findings highlight the presence of antimicrobial‐resistant bacteria in captive wildlife and underscore the need for strengthened biosecurity, prudent antimicrobial use and integrated One Health surveillance within wildlife facilities.

### 5.1 Recommendations

- The UWEC management and workers are encouraged to take care when handling the birds and tortoises to minimize the chances of acquiring bacteria that carry the genes coding for resistance. This can be done through wearing protective gear such as gloves.
- In addition, the authorities in charge of the animal industry should put a regular monitoring and surveillance system to track the AMR status at the facility. This will enable easy tracking and putting up the required regulations to curb AMR.
- Regularly screening species at UWEC for resistant isolates and genes.
- Use of more sensitive techniques in identification of bacteria.
- The centre is advised to use antibiotic classes interchangeably so as to minimize constant exposure of microorganisms to certain drugs.
- Use of sulphonamides should be minimal or excluded in the drugs of choice.

## Author Contributions

**Delilah Diana Namara** conducted field data collection, laboratory culturing and DNA extraction, and drafted the manuscript. **Paul Ssajjakambwe** supervised the project. **Sarah Robinah Nakabuye** and **Vanessa Alyj Katabazi** performed data analysis. **Celsus Sente** supervised data collection, laboratory work and manuscript development. All authors read and approved the final version of the manuscript.

## Funding

This study was partly funded by the Byamukama family.

## Ethics Statement

This study was reviewed and approved by the Research Panel of the College of Veterinary Medicine, Animal Resources and Biosecurity (COVAB), Makerere University, and subsequently by the Uganda Wildlife Conservation Education Centre (UWEC) Research Committee on 13 July 2022. The study was conducted retrospectively using samples collected as part of routine animal health screening. No additional animal handling or sampling was conducted specifically for this study; therefore, Institutional Animal Care and Use Committee (IACUC) approval was not required. Individual animal identifiers were removed during analysis. This study did not involve human participants, and Institutional Review Board approval was not applicable.

## Conflicts of Interest

The authors declare no conflicts of interest.

## Data Availability

The data used in this study are available from the corresponding author upon reasonable request and with permission from the Uganda Wildlife Conservation Education Centre (UWEC).
